# Saunders’ Medical Hand Atlases

**Published:** 1901-08

**Authors:** 


SAUNDERS' MEDICAL HAND ATLASES.—ATLAS AND EPITOME OF OBSTETRIC DIAGNOSIS AND TREATMENT. By Dr. O. Shaeffer, of Heidelberg. From the second revised German edition. Edited by J. Clifton Edgar, M. D., Professor of Obstetrics and Clinical Midwifery, Cornell University Medical School. With 122 colored figures on 56 plates, 38 other illustrations, and 317 pages of text. Philadelphia and London: W. B. Saunders & Co. 1901. Cloth, $3.00 net.

In addition to a large and unusually well executed collection of colored lithographic illustrations the book contains a text of great value, it being a complete and comprehensive abstract of obstetric diagnosis and treatment.

In the preparation of this work the author has been "guided chiefly by the demands of the practical, clinical side of obstetrics," and for this reason all that part of the text not likely to be of much immediate practical value to the obstetrician is printed in type smaller than the rest.

Practically all of the fifty-six plates and most of the other illustrations are worthy of the highest praise.

